# Paracentesis of an Ovarian Cyst During Second-Trimester Pregnancy

**DOI:** 10.7759/cureus.19610

**Published:** 2021-11-15

**Authors:** Dionysios Galatis, Nikolaos Kiriakopoulos, Ioannis Komiotis, Christos Benekos, Georgia Micha, Konstantina Kalopita, Ioannis Gripiotis, Antonios Kondylios, Christos Parthenis, Antonios Strongylos, Thalis Papapostolou, Argyrios Monastiriotis

**Affiliations:** 1 Fifth Department of Obstetrics and Gynecology, Helena Venizelou, General and Maternity Hospital, Athens, GRC; 2 Department of Anesthesiology, Helena Venizelou, General and Maternity Hospital, Athens, GRC; 3 Department of Anesthesiology, General and Maternity Hospital of Athens, Athens, GRC; 4 Sixth Department of Obstetrics and Gynecology, Helena Venizelou, General and Maternity Hospital, Athens, GRC; 5 First Department of Obstetrics and Gynecology, Helena Venizelou, General and Maternity Hospital, Athens, GRC

**Keywords:** torsion, pregnancy, paracentesis, ovarian cyst, cyst

## Abstract

A common issue is that modern obstetricians are required to manage ovarian cysts during pregnancy. Most lesions are benign and will spontaneously resolve, with a few exceptions. Management practices include conservative observation or surgery. Asymptomatic women with an ovarian cyst larger than 5 cm should undergo serial ultrasounds up to 16 weeks of pregnancy and, if the mass does not regress, further management with imaging or surgery is to be considered. This article presents a case of an ovarian cyst sized 21 cm in a second-trimester pregnancy and its management. Paracentesis was performed due to persisting symptoms. The procedure was performed with no complications for the mother and no adverse effects for the fetus. The patient was discharged in good health.

## Introduction

In antenatal examinations, the modern obstetrician is commonly confronted with ovarian cysts. Most of these lesions are benign and identified as corpus luteum cysts. They spontaneously regress during pregnancy, with only a few in need of surgical intervention [1]. Pelvic ultrasonography is the modality of choice to guide diagnosis and further management. It aids the diagnosis in cases of badly defined masses and the use of color Doppler imaging enables the distinction of malignant from benign masses [2]. Up to 20% of adnexal lesions that cannot be distinguished by ultrasound can be evaluated by using magnetic resonance imaging (MRI) [3]. The origin of the mass and its tissues can be accurately identified, in addition to evaluation of the stage of the invasion in possibly malignant disease. Tumor markers have drawbacks that exclude them from routine diagnostic practice. A high count of CA-125 nearing 10,000 U/mL, however, comprises a serious alert for possible malignancy [4], taking into account the fact that normal pregnancies rarely present CA-125 levels higher than 35 U/mL [5].

Endometriomas, teratomas, corpus luteum and theca lutein cysts present complex features on an ultrasound scan [6, 7]. Nevertheless, serous or mucinous cystadenomas can present as benign cysts [7]. According to the literature, the adnexal masses most commonly encountered during pregnancy are dermoid cysts and cystadenomas. The incidence of malignancy reaches up to 5% [4].

Ovarian masses can have adverse effects during pregnancy. Ovarian torsion, rupture of the cyst, malignancy or obstruction of labor are likely complications, related to the size and complexity of the mass [2]. Management practices include conservative observation or surgical intervention. The literature suggests that asymptomatic women with an ovarian cyst larger than 5 cm should undergo serial ultrasounds up to 16 weeks of pregnancy and, if the mass does not regress, further management with imaging or surgery is to be considered.

We present this case of an ovarian cyst sized 21 cm in a second-trimester pregnancy and its management.

## Case presentation

**Figure 1:**
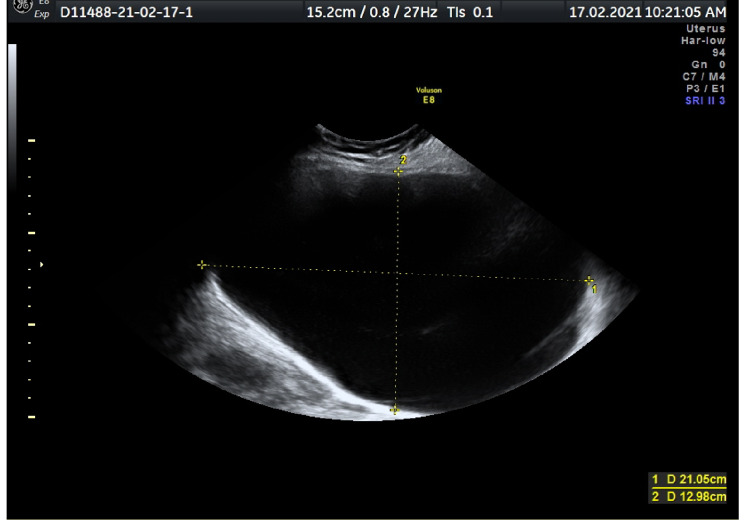
Ovarian cyst having size 21.05 x 12.98 cm

The patient, 33 years of age, presented with symptoms of distention and pain in the area of the lower abdomen and was admitted to the hospital with a diagnosis of G6P2 at 15 weeks’ gestation. She had had two previous miscarriages that did not reach 24 weeks of gestation and abortion for undisclosed reasons. Her medical history included G6PD deficiency. During antenatal care, a routine ultrasound scan at 7+1 weeks’ gestation showed an ovarian cyst on the right ovary, 4.08 x 3.39 cm in size. Subsequent ultrasound scans showed the progressive growth of the cyst. On a nuchal translucency scan at 12+1 weeks’ gestation, the cyst had reached 19 cm and, at the time of admission, the cyst had reached the size of 21.05 x 12.98 cm (Figure 1).

**Figure 2:**
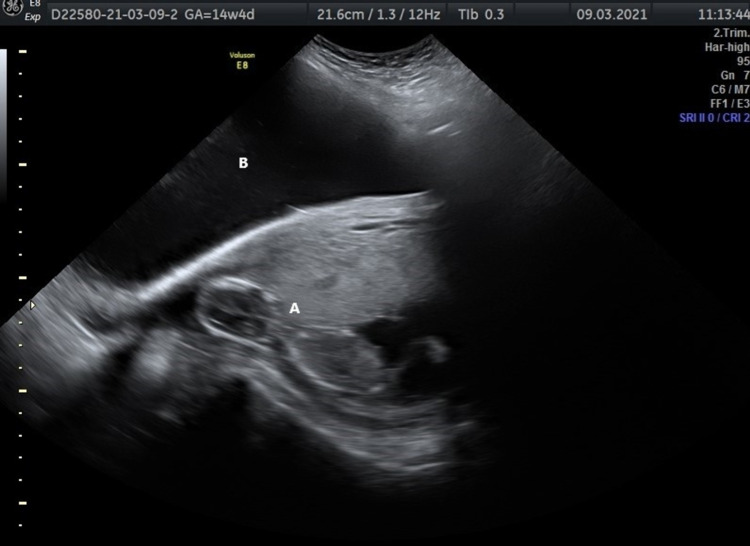
A indicates intrauterine fetus, 15 weeks' gestation and B indicates ovarian cyst

Basic clinical signs were measured upon admission. The patient’s blood pressure was 115/65 mmHg with a heart rate of 79 bpm. The patient's cardiac evaluation was normal. The cervix did not present with effacement or dilation. There were no symptoms of vaginal bleeding. The nuchal translucency scan did not present any abnormalities, apart from the ovarian cyst. The patient’s hematocrits were 33.2%, INR was 1.00 and she was blood type A negative. Rhogam was administered in her previous delivery. Screening for common viruses did not show any current infections. CA-125 marker was 14 U/mL. The ultrasound scan upon admission showed, apart from the ovarian cyst, an intrauterine pregnancy of one fetus (placenta in anterior position) at 15 weeks’ gestation (Figure 2), fetal heartbeat positive at 148 bpm and free fluid in the pouch of Douglas. The scan was performed with a model Voluson E8 Expert.

Transvaginal culdocentesis was performed on the patient. A clear, free fluid of 10 mL volume was collected and sent for extemporaneous cytological examination. The results of the examination were negative for malignancy.

**Figure 3:**
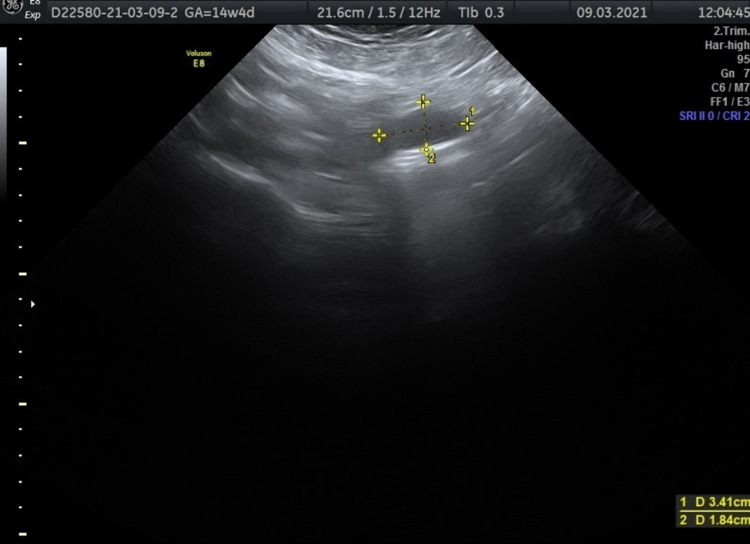
Ovarian cyst size 3.41 x 1.84 cm

Considering the appearance of symptoms related to the existence of the ovarian cyst and confirming the absence of malignancy, a paracentesis was planned. A Braun needle 18G x 3 ½” 1.3 x 88 mm was used to aspirate the fluid from inside the cyst. The total volume of aspirated serous fluid reached 3,540 mL. A sample of 290 mL was sent for cytological examination. Following the end of the paracentesis, a repeat ultrasound scan was performed. The ovarian cyst was drastically reduced in size, having a maximum diameter of 3.41 cm (Figure 3). No abnormalities were found regarding the fetus.

Post-operatively, cefoxitin 1g x 3, along with analgesics (paracetamol 1g x 3) and progesterone, tablet 400mg x 3, were administered to the patient for 24 hours. The post-operative condition of the patient was free of complications. The patient was deemed well and discharged the next day.

Fifteen days later, the patient was followed up with an ultrasound scan to ensure the viability of the pregnancy. No abnormal changes were found in the fetus. There were no signs of an ovarian cyst. A month later, the results of the cytological examination showed a serous fluid.

## Discussion

Adnexal masses present at a rate of incidence of 2.3% during pregnancy [8]. In the first trimester of pregnancy, the majority of ovarian cysts encountered are constituted of corpus luteum or other small functional cysts. These types of cysts typically regress spontaneously. It is documented that complications due to ovarian cysts occur in 10%-30% of pregnant women. The possibility of ovarian torsion can reach as high as 28% [8]. The most common period for ovarian torsion is the first trimester of pregnancy, reaching up to 68% [9].

Additionally, ovarian cysts pose a risk of dystocia. A study on this subject found that 25% of patients in whom an adnexal mass was identified had a cesarean delivery due to labor dystocia [6].

Management of ovarian cysts during pregnancy has strong advocators for both conservative follow-up measures and surgical intervention. Neither method is free of risks. Conservative management involves the possibility of ovarian torsion that is not common following laparoscopy [4]. On the other hand, surgical laparoscopy carries obstetric complications, such as spontaneous abortion, low birth weight, preterm delivery and the use of tocolytics for preterm labor, fetal anomalies and low Apgar score [10], conditions that probably would cause the mother to abstain from this procedure. The recommendations indicate that the optimum window for surgical management is 16 to 20 weeks’ gestation for laparoscopy [2].

An alternative technique is paracentesis of the cyst with a needle and drainage of its contents under sonographic guidance. This technique has been associated with uncontrolled bleeding, but there is an extremely small percentage of recurrence of ovarian torsion [11]. In the hands of an experienced practitioner, the procedure can produce safe results, with no adverse maternal or fetal effects. With that in mind, more studies in this area would present the clear benefits of this alternative treatment method, as well as establish the optimum window of gestational age for the paracentesis.

In our case, the decision to perform paracentesis of the ovarian cyst in the patient was made on the grounds of its presenting symptoms. The aspiration was performed with no adverse effects to the fetus and the patient showed significant improvement of the presenting symptoms. The patient was discharged the next day in good health.

## Conclusions

Current data are inconclusive on the best management practices for adnexal masses during pregnancy, advocating either surgery at the best possible moment or conservative measures. This article presented an alternative method of management of ovarian cysts, paracentesis with a needle and drainage under sonographic guidance. It is clear that further studies are needed to guide the diagnostic modalities and optimal management options. All methods have their merits and, choosing the correct one in each case, can lead to a favorable outcome.
